# A Series of Severe and Critical COVID-19 Cases in Hospitalized, Unvaccinated Children: Clinical Findings and Hospital Care

**DOI:** 10.3390/epidemiologia6030040

**Published:** 2025-08-04

**Authors:** Vânia Chagas da Costa, Ulisses Ramos Montarroyos, Katiuscia Araújo de Miranda Lopes, Ana Célia Oliveira dos Santos

**Affiliations:** 1 Postgraduate Program in Health Sciences, Universidade de Pernambuco, Recife 50100-130, Brazil; ulisses.montarroyos@upe.br (U.R.M.); ana.oliveira@upe.br (A.C.O.d.S.); 2 School of Nursing Our Lady of Grace, Universidade de Pernambuco, Recife 50100-130, Brazil; katiuscia.lopes@upe.br; 3 Street Arnóbio Marques, 310 Santo Amaro, Recife 50100-130, Brazil

**Keywords:** COVID-19, infectious diseases, children, hospitalization, patient severity, hospital care, medical tests

## Abstract

Background/Objective: The COVID-19 pandemic profoundly transformed social life worldwide, indiscriminately affecting individuals across all age groups. Children have not been exempted from the risk of severe illness and death caused by COVID-19. Objective: This paper sought to describe the clinical findings, laboratory and imaging results, and hospital care provided for severe and critical cases of COVID-19 in unvaccinated children, with or without severe asthma, hospitalized in a public referral service for COVID-19 treatment in the Brazilian state of Pernambuco. Methods: This was a case series study of severe and critical COVID-19 in hospitalized, unvaccinated children, with or without severe asthma, conducted in a public referral hospital between March 2020 and June 2021. Results: The case series included 80 children, aged from 1 month to 11 years, with the highest frequency among those under 2 years old (58.8%) and a predominance of males (65%). Respiratory diseases, including severe asthma, were present in 73.8% of the cases. Pediatric multisystem inflammatory syndrome occurred in 15% of the children, some of whom presented with cardiac involvement. Oxygen therapy was required in 65% of the cases, mechanical ventilation in 15%, and 33.7% of the children required intensive care in a pediatric intensive care unit. Pulmonary infiltrates and ground-glass opacities were common findings on chest X-rays and CT scans; inflammatory markers were elevated, and the most commonly used medications were antibiotics, bronchodilators, and corticosteroids. Conclusions: This case series has identified key characteristics of children with severe and critical COVID-19 during a period when vaccines were not yet available in Brazil for the study age group. However, the persistence of low vaccination coverage, largely due to parental vaccine hesitancy, continues to leave children vulnerable to potentially severe illness from COVID-19. These findings may inform the development of public health emergency contingency plans, as well as clinical protocols and care pathways, which can guide decision-making in pediatric care and ensure appropriate clinical management, ultimately improving the quality of care provided.

## 1. Introduction

The COVID-19 pandemic took the world by surprise, from its initial emergence in December 2019 in Wuhan, China, to the rapid global spread, and the severe indiscriminate impact it had on all age groups. The resulting loss of life profoundly affected tens of thousands of individuals, fundamentally affecting lives over the course of more than three years. Even now, the global incidence of COVID-19 remains a significant public health problem, with ongoing prevalence and lingering symptoms continuing to compromise the health of populations worldwide [1].

In May 2023, the World Health Organization (WHO) declared the end of the pandemic. However, due to the emergence of new strains with further mutations in the Spike protein, the WHO issued a strategic transition plan to shift from emergency status to long-term management of COVID-19 [2]. This plan includes guidelines for prevention, control, and disease management [3].

Brazil maintains surveillance of the epidemiological scenario for detection and identification of respiratory viruses present in cases of severe acute respiratory syndrome (SARS) through molecular tests RT-PCR (Reverse-Transcriptase Polymerase Chain Reaction). Of the 36,097 cases of SARS in all age groups, reported in the epidemiological year 2025, 41.2% were positive for some respiratory virus; of these, 26% were positive for SARS-CoV-2. During the same period, in all age groups, 2330 deaths from SARS were recorded, with 71.9% of these attributed to SARS-CoV-2, while 29.1% died of other respiratory viruses, such as rhinovirus, influenza, and respiratory syncytial virus [4]

As of March 2025, Brazil ranks sixth globally in the number of COVID-19 cases reported to WHO, reported 37.3 million cases, and second in cumulative COVID-19-related deaths. The United States and China are the countries with the highest number of cases reported cumulatively to the WHO, with 103 million and 99.4 million cases reported, respectively. This underscores the continued circulation of SARS-CoV-2 and its variants, which continue to infect individuals across all age groups, even after the official end of the pandemic [1].

Throughout the COVID-19 pandemic, SARS-CoV-2 affected children across a wide spectrum of disease severity. Although the majority of cases presented asymptomatically or with mild symptoms, many children experienced severe illness [5].

This broad clinical spectrum of severity is also heterogeneous, potentially manifesting as SARS, myocarditis and cardiovascular alterations, thromboembolism and deep vein thrombosis, acute or subacute neurological conditions, acute febrile syndromes, acute abdominal presentations, and multisystem inflammatory syndrome in children (MIS-C), temporarily associated with COVID-19. It may also destabilize the management of chronic conditions common in childhood, such as type 1 diabetes [6]. Severe cases of COVID-19 require hospital admission and specific treatment, including antiviral therapy and supportive clinical measures such as oxygen therapy through invasive and non-invasive devices, mechanical ventilation, continuous medication infusion, and vital sign monitoring.

Vaccination is recommended to protect against COVID-19 and to achieve herd immunity across all age groups [7]. However, data from the Brazilian Ministry of Health have revealed low vaccination coverage among children. As of April 2025, 35.98% of children aged 6 months to 2 years had received only two doses of the vaccine, while just 11.34% had completed the full three-dose regimen. Among children aged 3 to 4 years, 30.81% had received two doses, and only 15.33% had full coverage with all three doses. In the 5 to 11-year age group, 60.52% had received two doses, while 23.89% had completed the three-dose series [8].

This scenario of low vaccination coverage, combined with the ongoing incidence of SARS caused by SARS-CoV-2, poses significant challenges to the effective control of COVID-19. In this context, examining the clinical, laboratory, and imaging findings in pediatric COVID-19 cases, as well as the therapeutic requirements for hospital care during a period when vaccines were not yet available for this age group, offers important insight into the impact of the disease on children. Despite the current availability of vaccines, coverage remains insufficient, largely due to parental hesitancy.

This study aimed to describe the clinical presentations, laboratory and imaging findings, and hospital care provided in severe and critical cases of COVID-19 among unvaccinated children, with or without severe asthma, hospitalized at a public referral center for COVID-19 treatment in the northeastern Brazilian state of Pernambuco.

## 2. Materials and Methods

Study type: Case series of severe and critical COVID-19 in hospitalized, unvaccinated children, with or without severe asthma.

Study site: Oswaldo Cruz University Hospital at the Universidade de Pernambuco, a referral center for infectious diseases and epidemiological risk, and a member of committees responsible for developing care protocols in epidemic situations. In March 2020, 53 intensive care unit (ICU) beds and 133 ward beds for adult and pediatric patients were designated exclusively for COVID-19 treatment.

Participants: The study included all children aged from birth to under 12 years who were hospitalized with COVID-19, with or without severe asthma. In accordance with the Child and Adolescent Statute (ECA), a child is defined as an individual under the age of 12.

Inclusion and exclusion criteria: The study included children with laboratory-confirmed severe and critical COVID-19, diagnosed by RT-PCR, the gold standard test recognized by WHO, with or without severe asthma, regardless of their nutritional status. Children with other comorbidities, including oncological diseases, genetically inherited disorders, genetic syndromes, chronic encephalopathies, congenital heart diseases, and HIV exposure, were excluded from the study.

Data collection: The information was collected from secondary data. Initially, a list was compiled of all children hospitalized in the pediatric wards, inpatient units, and pediatric intensive care units (PICU) between March 2020 and June 2021, prior to the commencement of vaccination for the study population. Using the Laboratory Environment Manager (GAL) system from the Pernambuco Health Secretariat, all children with a positive RT-PCR test for SARS-CoV-2 were identified. Hospital admission records were then reviewed, and relevant information regarding clinical progression and hospital care was extracted from medical, nursing, and physiotherapy records, as well as laboratory and imaging test results. A data collection form developed by the researchers was used to organize this information.

Studied variables include the following.

Sociodemographic variables: age, sex, municipality of residence.

Clinical and care variables: weight, height, other medical diagnoses, medication therapy (antibiotics, antivirals, corticosteroids, bronchodilators, vasopressors, sedatives, muscle relaxants, diuretics, antihypertensives); invasive and non-invasive ventilatory support; blood transfusions; laboratory test results such as lymphocyte count, C-reactive protein (CRP), D-dimer, lactate dehydrogenase (LDH), alanine aminotransferase (ALT), aspartate aminotransferase (AST), CKMB, CPK, troponin, and ferritin; and imaging test results such as chest X-ray, chest and abdominal ultrasound, chest CT scan, and echocardiogram. The nutritional status was categorized according to weight ranges from the WHO physical growth curves for children, including underweight, normal weight, and overweight/obesity, calculated using the Anthro 3.2.2 software (children aged 0–5 years) and AnthroPlus 1.0.4 software (children aged 5–19 years) developed by the WHO. The comorbidity variable was as follows: severe asthma—continuous/daily use of two inhaled control/preventive medications, or omalizumab or oral corticosteroids OR asthma with hospitalization in the last 12 months.

The classification of severe and critical COVID-19 cases follows the criteria established by the Brazilian Ministry of Health. Severe COVID-19 is defined as SARS, characterized by flu-like symptoms presenting with dyspnea/respiratory distress or persistent chest pressure, or oxygen saturation below 95% in ambient air, or bluish-color lips or face. In children, key symptoms include tachypnea (≥70 breaths per minute for infants under 1 year and ≥50 breaths per minute for children over 1 year), hypoxemia, respiratory distress, altered consciousness, dehydration, difficulty feeding, myocardial injury, elevated liver enzymes, coagulation dysfunction, rhabdomyolysis, central cyanosis, or SpO2 <90–92% at rest in ambient air, lethargy, seizures, and difficulty/refusal to feed.

Critical COVID-19 includes the main symptoms of sepsis, acute respiratory distress syndrome, severe respiratory failure, multiple organ dysfunction, severe pneumonia, need for respiratory support, and ICU admissions.

The total duration of hospital admission was measured in days, including the time spent in other healthcare units for COVID-19 treatment prior to being transferred to the study site, as well as the time in the inpatient ward and/or PICU. The outcome was defined as either hospital discharge or death.

Data Analysis: The database was managed using MS Office Excel, and statistical analysis was performed using Jamovi 2.4. Continuous variables were tested for normality of distribution using the Shapiro–Wilk test. Variables with non-normal distribution were described using median and interquartile range. Categorical variables were described as proportions.

## 3. Results

A total of 1006 children were hospitalized in the inpatient ward and/or PICU from March 2020 to June 2021 at the study site with a diagnosis of COVID-19. Of these, 837 cases confirmed through clinical epidemiological criteria and immunological testing were excluded. Among the 169 cases confirmed through laboratory criteria with a positive RT-PCR test for SARS-CoV-2, 66 were excluded due to comorbidities, including oncological diseases, hereditary conditions caused by genetic mutations, genetic syndromes, chronic encephalopathies, congenital heart diseases, and HIV exposure. Additionally, mild (n = 18) and moderate (n = 5) cases were also excluded. The final case series consisted of 80 children aged from 1 month to 11 years, diagnosed with severe and critical COVID-19, with no comorbidities, with severe asthma, and with nutritional status classified as underweight, normal weight, and overweight/obesity. Regarding the outcome, 79 (99.5%) of the cases were discharged, with one death (0.5%) observed. The sociodemographic characteristics and other diagnostic hypotheses for hospital admission are detailed in Table 1.

Biochemical tests were used to screen for complications related to the systemic inflammatory process. Table 2 presents the altered findings that outline the systemic inflammatory profile associated with SARS-CoV-2 infection, based on laboratory tests requested according to the child’s clinical condition.

Imaging exams were performed according to the clinical progression of the child. The most frequent findings on chest X-rays were bilateral diffuse infiltrates, interstitial infiltrate in the paracardiac region, perilobar infiltrate, and perihilar infiltrate, which represented 55% of the alterations. Ground-glass opacity was identified in one chest X-ray and in eight chest CT scans. Pleural effusion, identified by chest CT scans in seven cases, was corroborated by chest ultrasound (USG) images in three of these cases. Children with cardiac and gastrointestinal involvement, evidenced by echocardiogram and abdominal USG, were diagnosed with MIS-C. The alterations in the imaging exams are described in Table 3.

Throughout hospitalization, the children received hospital care tailored to their clinical progression. Although all met the criteria for classification as severe or critical cases, their treatment included supportive measures, medication treatment, and blood therapy adjusted according to the deterioration of their clinical condition.

Some children required multiple modalities of oxygen therapy, initially using non-invasive devices such as oxygen catheters and non-rebreathing masks (NRM), and invasive devices such as orotracheal tube and tracheostomy, which were used when respiratory distress intensified.

The medication treatment included antibiotics (azithromycin, ceftriaxone, vancomycin, among others), bronchodilators (salbutamol), corticosteroids (dexamethasone, hydrocortisone, prednisolone, methylprednisolone), antivirals (oseltamivir), sedatives (midazolam, fentanyl), and vasoactive drugs (adrenaline, norepinephrine, dopamine, dobutamine), anticoagulants (enoxaparin sodium), and diuretics (furosemide), which were the most commonly prescribed drugs. The hospital care and medicinal treatment are described in Table 4.

Of the children in this case series, 46.2% (n = 37) had been previously hospitalized in other healthcare services and were transferred to the referral center, the study site. A percentage of 33.7% (n = 27) of the children required hospitalization in the pediatric intensive care unit. The duration of hospitalization in the PICU ranged from a minimum of one day to a maximum of 63 days, and the total hospital stay ranged from a minimum of one day to a maximum of 140 days. The medians of the total hospital stay are described in Table 5.

## 4. Discussion

This case series of severe and critical COVID-19 in hospitalized, unvaccinated children has demonstrated that the most affected age group was children aged under two years, predominantly male, primarily presenting with respiratory illnesses, presence of chronic diseases such as severe asthma and overweight/obesity, and the need to use oxygen therapy at hospital admission. Chest X-ray, a low-cost imaging test and routinely performed in emergencies, is able to identify lesions suggestive of pulmonary impairment by SARS-CoV-2.

Several studies [7,9,10] have similarly reported a higher frequency among males, even when sex-based differences are relatively small. A multicenter cohort study [11] also observed a predominance of male patients, with percentages comparable to those in our findings. These results reinforce the notion of a protective factor associated with the female sex and underscore the increased disease severity in younger children, and those hospitalized due to respiratory illnesses.

Respiratory conditions, such as viral bronchiolitis and pneumonia, are among the most frequently observed [6]. Although respiratory diseases accounted for 73.8% of the sample in this study of critically ill children—a proportion justified by the predominant respiratory involvement characteristic of COVID-19—children with gastrointestinal pathologies resulting from SARS-CoV-2 infection also progressed to severe illness. Gastrointestinal symptoms have been associated with severe and critical cases of COVID-19 in children [12]. These findings highlight the importance of not limiting clinical attention to respiratory symptoms alone; gastrointestinal complaints, even in the absence of respiratory symptoms, may also signal SARS-CoV-2 infection in children.

This is attributable to the fact that COVID-19 is a disease with systemic manifestations. In addition to the respiratory tract, the gastrointestinal tract, heart, and kidneys also express angiotensin-converting enzyme 2 (ACE2), the receptor for the SARS-CoV-2 Spike protein, which is involved in regulating extracellular fluid volume and blood pressure [13,14].

Children with comorbidities became a source of concern for both parents and healthcare providers, as chronic conditions were suspected to increase the risk of severe COVID-19—a concern later substantiated as the pandemic progressed. Scientific evidence has demonstrated that certain comorbidities, particularly severe asthma and the chronic inflammatory state linked to obesity, increase the likelihood of severe COVID-19 in pediatric populations. In this case series, 11.2% of severe and critical cases involved children with overweight or obesity, reinforcing findings that identify obesity as a predictor of COVID-19 severity in children [11,15].

Similarly, 17.5% of cases presented with severe asthma, aligning with previous studies [15] that associate this condition with increased disease severity and a higher risk of admission to PICU [16]. Notably, 17% of children with severe asthma were also overweight or obese. The baseline inflammatory state exacerbated by obesity, observed in cases of non-allergic asthma, further increases susceptibility to severe COVID-19 outcomes [17].

Although the presence of comorbidities indicates a higher risk of severe clinical outcomes, this case series has illustrated that children with no comorbidities also developed severe illness [11,12].

Multisystem inflammatory syndrome in children represents a severe, delayed immune response to SARS-CoV-2 infection observed in children and adolescents [18]. It is characterized by elevated levels of inflammatory markers, particularly C-reactive protein (CRP), D-dimer, and erythrocyte sedimentation rate (ESR), as well as coagulopathy and organ dysfunction [19]. In this case series, 15% of patients met the diagnostic criteria for MIS-C, all of whom exhibited abnormalities in at least one inflammatory marker. Lymphocytopenia and higher CRP levels are recognized as predictive indicators of MIS-C [11].

The systemic inflammatory process is evidenced by lymphocytopenia and increased inflammatory and cardiac markers. This intense inflammatory response is associated with the severity of COVID-19, affecting multiple systems due to immune dysregulation, which manifests in the clinical progression of severe pediatric cases [20]. High levels of CRP, ferritin, D-dimer, AST, ALT, and LDH, among others, have been associated with severe COVID-19. Notably, LDH has been identified as a predictor that significantly increases the risk of severe COVID-19-related pneumonia [21].

Imaging findings from chest X-rays, thoracic ultrasound, chest CT scans, and echocardiography were consistent with COVID-19-related involvement [22,23,24]. The presence of pulmonary infiltrates on chest X-rays is considered a predictor of disease severity in COVID-19 [11]. In our case series, infiltrates were identified in 55% of the chest X-rays. Among children who developed acute respiratory failure, imaging revealed bilateral diffuse pulmonary infiltrates [25].

Cardiac involvement, as documented by echocardiography in five children, included pericardial effusion, ventricular dysfunction, myocardial dysfunction, and myocardial infarction. Cardiovascular complications are recognized as significant outcomes of SARS-CoV-2 infection in children [26] and, in this series, were associated with MIS-C.

Hypoxia on hospital admission has been described as a predictor of severe disease [11]. In this case series, 65% of children required oxygen therapy, delivered through both invasive and non-invasive devices.

Admission to a PICU reflects a deteriorating clinical condition and the need for advanced life-support measures. In this series, 33.75% of patients required PICU admission at some point during hospitalization. Given that the demand for PICU beds often exceeds availability, admission is generally reserved for the most critically ill children. The length of stay is determined by the trajectory of clinical improvement and the stabilization of hemodynamic and respiratory parameters.

Approximately 15% of the children progressed to respiratory failure and required invasive mechanical ventilation. Two of these patients required prolonged ventilatory support and subsequently underwent tracheostomy. These findings are comparable to those reported in the cohort study by Fernandes et al. (2021) [11], in which 41% of the pediatric patients were admitted to the PICU and 18% required invasive mechanical ventilation.

The use of vasoactive drugs to maintain hemodynamic stability, sedatives to facilitate adaptation to invasive mechanical ventilation, certain classes of antibiotics, blood transfusions, and nutritional support via gastric or enteral feeding tubes all require continuous infusion through advanced medical technologies. These specialized care requirements reflect the severity of the disease course in affected children [25].

The risk of respiratory failure requiring mechanical ventilation, as well as circulatory failure necessitating the use of vasoactive drugs, is correlated with laboratory abnormalities. These include high levels of ferritin and D-dimer, the lymphocyte count, complete blood count parameters, hematocrit, troponin, and platelet count, among others [27].

At the onset of the pandemic, antibiotics were widely used under the assumption of bacterial superinfection [28], as reflected in our findings, where 88.8% of children had received at least one antibiotic. Most had been treated with up to three antibiotics simultaneously, and treatment regimens were often modified due to persistent fever. Other studies similarly reported antibiotics as the most frequently prescribed medications, with usage rates of 88.2% [29] and 77% [30] among children.

The urgency to identify effective treatments for COVID-19 prompted the repurposing of existing medications [31]. Among them, bronchodilators were reused due to their anti-inflammatory and bronchodilatory properties, which form the cornerstone of treatment for inflammatory pulmonary diseases such as asthma and chronic obstructive pulmonary disease (COPD) [32]. In this case series, a β2-adrenergic receptor agonist bronchodilator, salbutamol, was administered to 63.7% of the children. A previous study [33] reported that salbutamol improved pulmonary function and supported respiratory recovery in patients with moderate to severe COVID-19.

Corticosteroids were administered to 57.5% of the children. These agents are widely used to suppress inflammatory processes [34], and a study [35] demonstrated that dexamethasone administration reduced mortality by 50% in patients with severe COVID-19.

In this case series, 99.5% of the children were discharged from the hospital, a favorable outcome that aligns with findings from other studies reporting positive prognoses in children with severe and critical COVID-19, even among those with persistent symptoms [36].

The availability of vaccines for the pediatric population represents a promising strategy for controlling COVID-19, since prior immunity offers protection against the severe sequelae of SARS-CoV-2 infection [37]. However, in Brazil, vaccine coverage among children aged 5 to 11 years has remained low since the initiation of the vaccination campaign since the start of the COVID-19. In 2022, only 69.5% of children received the first dose, and just 46.1% received the second dose. Several factors have contributed to this low coverage, including socioeconomic inequalities, geographic barriers, limited access to and availability of healthcare services, and parental vaccine hesitancy [38].

Although the COVID-19 vaccine was included in Brazil’s National Immunization Program in January 2024 for children aged 6 months to under 5 years [39], with the first dose administered at 6 months, the second at 7 months, and a third dose at 9 months for priority groups, vaccination coverage has remained insufficient. This ongoing gap continues to contribute to illness and death among children [9]. Despite the official declaration of the COVID-19 pandemic, the emergence and circulation of new SARS-CoV-2 variants have perpetuated the prevalence of the disease, including cases of prolonged symptoms [40].

The selection of participants based solely on laboratory-confirmed SARS-CoV-2 infection may be considered a limitation of this case series, primarily due to constraints in sample size. Nonetheless, the findings provide an accurate representation of the clinical demands and hospital care required for children with severe forms of COVID-19. Being a retrospective study, conducted in a single hospital, of the initial period of infections in children, information on SARS-CoV-2 variants was not available.

## 5. Conclusions

This case series involving hospitalized children has identified key clinical, laboratory, and imaging characteristics associated with severe and critical COVID-19 during a period in which vaccines were not yet available for this age group in Brazil. The findings also underscore the specialized hospital care required in such cases, including access to both non-invasive and invasive oxygen therapy, mechanical ventilation, technological support for the continuous infusion of medications such as vasoactive drugs and sedatives, and the availability of pediatric intensive care unit beds.

Although the COVID-19 pandemic officially ended after more than three years, respiratory infections continue to affect children seasonally, caused by a range of viruses, including SARS-CoV-2 and its variants. Despite the availability of the COVID-19 vaccine, persistently low vaccination coverage, largely driven by parental hesitancy, contributes to continued vulnerability of children to severe forms of the disease.

These findings contribute to the body of knowledge on COVID-19 in children and its severity-related aspects, and may inform the development of public health emergency contingency plans, as well as the design of care protocols and service workflows. Such measures can support evidence-based clinical decision-making, ensuring that pediatric patients receive appropriate needs-based clinical management and that high-quality care is delivered across all levels of the pediatric healthcare system.

Based on our findings, we recommend that further research be conducted in healthcare settings similar to ours, as well as in settings where vaccination coverage is already established, so that new insights can contribute to knowledge about the severity of COVID-19 in children.

## Figures and Tables

**Table 1:** Distribution of sociodemographic characteristics and other diagnostic hypotheses related to hospital admission in unvaccinated children with severe and critical COVID-19 (n = 80).

Clinical and Sociodemographic Characteristics	Number(%)
Age group	
Infant (1 month to 2 years)	47 (58.8)
Child (3 to 5 years)	8 (10.0)
Child (6 to 11 years)	25 (31.2)
Sex	
Male	52 (65.0)
Female	28 (35.0)
Nutritional status	
Underweight	4 (5.0)
Normal weight	67 (83.8)
Overweight/Obese	9 (11.2)
Distribution per region Pernambuco	
Recife	28 (35.0)
Metropolitan Area	25 (31.3)
Zona da Mata Region	6 (7.5)
Agreste Region	16 (20.0)
Hinterland	5 (6.2)
Other diagnostic hypotheses related to hospitalization	
Pneumonia/Bronchiolitis/Respiratory tract infection	45 (56.3)
Severe Asthma	14 (17.5)
* MIS-C	12 (15.0)
Gastrointestinal diseases	4 (5.0)
Others	5 (6.2)

* Multisystem inflammatory syndrome in children temporarily associated with COVID-19.

**Table 2:** Alterations in laboratory tests related to the systemic inflammatory profile of unvaccinated children with severe and critical COVID-19, (n = 80).

Variables	Number (%)
Lymphopenia	15 (30.6)
High levels of D-dimer	6 (31.6)
High levels of C-reactive protein (CRP)	38 (79.2)
High levels of lactate dehydrogenase (LDH)	22 (62.9)
High levels of aspartate aminotransferase (AST)	11 (27.5)
High levels of alanine aminotransferase (ALT)	8 (20.0)
High levels of cardiac markers (CPKMB, CPK, and troponin)	13 (52.0)
High levels of ferritin	19 (54.3)

**Table 3:** Alterations in imaging exams of hospitalized and unvaccinated children with severe and critical COVID-19.

Imaging Exams	Number of Exams Performed	Number of Exams with Alterations (%)	Alterations Encountered
Chest X-ray	56	40 (71.4)	Infiltrates, pleural effusion, hypotransparency, congestion, enlargement of the cardiac area, atelectasis, consolidation, condensation, ground-glass opacity.
Chest CT scan	11	11 (100)	Ground-glass opacity, pleural effusion, atelectasis, consolidation, congestion, pulmonary abscess.
Echocardiogram	7	5 (71.4)	Pericardial effusion, ventricular dysfunction, myocardial dysfunction, myocardial infarction.
Ultrasound	11	10 (90.9)	Chest: pleural effusion, atelectasis, pulmonary consolidation, sequela of deep venous thrombosis in the subclavian vein, hyperechoic blurring of deep adipose tissue in the clavicular region, axillary, and subclavian vein. Abdomen: hepatosplenomegaly, hepatomegaly, ascites, gallbladder distension, micro calculi in the gallbladder, lymph nodes in the flanks and periumbilical region.

**Table 4:** Hospital care and medicinal treatment in unvaccinated hospitalized children with severe and critical COVID-19 (n = 80).

Variables	Number (%)
Oxygen therapy	52 (65.0)
Non-invasive and invasive oxygen therapy devices:	
O2 Catheter	46 (57.5)
NRM	21 (26.3)
IMV * via OT **	10 (12.5)
IMV * via TQT ***	2 (2.5)
Venous Catheter Devices	
Peripheral venous catheter	63 (78.8)
Central venous catheter	15 (18.8)
Gastric tubing devices:	
Nasogastric/Nasoenteral tube	14 (17.5)
Medication Treatment	
Antibiotics	71 (88.8)
Bronchodilators	51 (63.7)
Corticosteroids	46 (57.5)
Antiviral (Oseltamivir)	24 (30.0)
Vasopressor	11 (13.8)
Sedatives	9 (11.3)
Muscular relaxants	3 (3.8)
Diuretics	8 (10.0)
Antihypertensives	3 (3.8)
Anticoagulant	2 (2.5)
Hemotherapy	
Packed red blood cells	7 (8.8)

* IMV = invasive mechanical ventilation; ** TOT = orotracheal tube; *** TQT = tracheostomy.

**Table 5:** Median hospital stay for hospitalized and unvaccinated children with severe and critical COVID-19, (n = 80).

Variables	Med (IQR)
Previous hospital stay (days)	3.0 (2.0–5.0)
ICU stay (days)	4.0 (1.5–7.0)
Ward stay (days)	5.0 (3.0–7.0)
Total hospital stay (days)	7.0 (5.0–12.0)

## Data Availability

The data presented in this study are available on request from the corresponding author due to the privacy of data.
